# Optimization of the Immersion Chemical-Mechanical Polishing Process for Gear

**DOI:** 10.3390/mi17070768

**Published:** 2026-06-24

**Authors:** Jian Sun, Longxing Liao, Fuli Cai, Mengqiao Guan

**Affiliations:** 1 College of Artificial Intelligence and Big Data, Luzhou Vocational and Technical College, Luzhou 646000, China; 2 College of Marine Equipment and Mechanical Engineering, Jimei University, Xiamen 361021, China15937473032@163.com (M.G.)

**Keywords:** gears, immersed chemical-mechanical polishing, FLUENT simulation, process optimization

## Abstract

To address surface quality defects caused by traditional mechanical polishing of gears, such as machining scratches and large surface waviness, this study proposes a novel immersed chemical-mechanical polishing (CMP) process for gear finishing. Numerical simulations were conducted in FLUENT to analyze the gear surface stress distribution and polishing fluid flow trajectories under different process conditions. The Euler–Euler method and RNG k–ε turbulence model were used to optimize process parameters and clarify the formation mechanism of ultra-smooth tooth surfaces. Experimental results for spiral bevel gears show that the proposed immersed CMP process effectively improves surface quality. The tooth profile roughness was reduced from Ra 1.531 μm to 0.509 μm, and surface scratches were significantly alleviated. These results confirm the feasibility and effectiveness of the proposed process. This study provides a reliable approach for efficient and precision polishing of complex-structured gears and extends the application of CMP technology to non-planar mechanical components.

## 1. Introduction

As the core basic components of mechanical transmission systems, gears are widely used in high-end equipment manufacturing fields such as automobiles, machine tools, aerospace, and marine engineering. Their surface machining quality directly determines the transmission efficiency, operating noise, wear resistance, and service life of the whole machine [1,2,3]. With the development of high-end equipment toward high speed, heavy load, and low noise, the requirements for gear surfaces have been upgraded from conventional precision machining to “ultra-low roughness, damage-free, and high consistency”. For example, aero-engine gears require a surface roughness Ra ≤ 0.2 μm with no residual stress or micro-cracks on the tooth surfaces [4,5].

The 40Cr steel has become a typical structural material for mid- to high-end gear manufacturing due to its high strength, good toughness, and excellent work-hardening properties. However, gears are characterized by complex tooth profiles and significant variations in tooth surface curvature, as well as poor machinability in the root and tip regions. Traditional machining processes struggle to balance machining efficiency and surface quality, which has become a core bottleneck restricting their application in high-end fields [5,6].

At present, precision machining technologies for gear surfaces are mainly divided into two categories: fixed abrasive machining and free abrasive machining [6,7,8,9,10,11,12,13]. Among fixed abrasive machining methods, CNC finish milling and grinding are the most widely used pre-finishing processes, which can accurately control the dimensional accuracy of gears. Nevertheless, the rigid contact between the cutting tool and the tooth surface during machining tends to cause defects such as grinding burns, large tooth surface waviness, and residual tensile stress. Moreover, tool interference is likely to occur for complex tooth profiles such as spiral bevel gears, resulting in poor consistency of tooth surface machining [9,10,14]. For instance, Yang et al. [10] proposed a CNC generating gear grinding method, which controls the grinding texture by adjusting the additional installation angle of the grinding wheel and achieves a surface roughness of approximately Ra 0.4 μm.

Free abrasive machining has become a research hotspot in precision polishing of complex-structured gears owing to its flexible machining characteristics. Abrasive flow polishing uses high-pressure fluid carrying abrasives to scour the tooth surface, enabling full-area coverage of the tooth surface. However, it suffers from limitations such as low material removal efficiency and easy deviation of tooth profile accuracy [15,16]. Vibratory finishing and barrel finishing realize impact cutting between abrasives and tooth surfaces through low-frequency vibration or workpiece rotation. Although they can reduce surface roughness, machining traces are difficult to completely eliminate, and the polishing time is relatively long [17,18,19,20]. Magnetorheological polishing and electrochemical polishing can achieve ultra-low damage machining, but magnetorheological polishing equipment is costly, and electrochemical polishing tends to cause uneven corrosion of the tooth surface, making it difficult to realize industrial batch application [21,22].

The above studies show that there are many precision machining methods for gears at present, which can meet the application requirements of most gears. However, the existing gear polishing processes have poor versatility and are mainly based on forced material removal by mechanical stress, resulting in common problems such as high residual stress, obvious machining traces, and large surface waviness. Therefore, it is urgent to explore a new gear polishing process.

Chemical-mechanical polishing (CMP) integrates the synergistic mechanism of chemical corrosion and mechanical micro-cutting. By selectively softening the workpiece surface via chemical reactions and gently abrading it with abrasive particles, CMP enables ultra-low-damage and ultra-smooth surface finishing, and has become a key technology for ultra-precision machining of planar components such as semiconductor wafers and optical elements [23,24,25]. In recent years, researchers have begun exploring the application of CMP to non-planar components. Luo et al. [26] employed a novel micro-nano bubble-enhanced immersed CMP method and achieved a surface roughness Sₐ of 3.913 nm on 316L stainless steel orthopedic implants. Wang et al. [27] proposed a combined process of ion beam figuring (IBF) and CMP for fabricating Montel mirrors used in laboratory X-ray systems, realizing high-precision and high-efficiency machining with a final surface form error of 3.82 nm RMS. Nevertheless, for complex curved parts such as gears, conventional CMP still faces technical bottlenecks, including the difficulty in uniformly applying polishing pressure and insufficient contact between slurry and tooth surfaces. The corresponding process system and parameter optimization methodology remain immature [28]. Most existing studies focus on CMP for planar or simple curved surfaces. Given the large curvature variation and high multi-region adaptability requirements of gear tooth surfaces, dedicated process design and simulation optimization methods are still lacking.

In addition, recent studies have shown that surface texture significantly influences fluid flow behavior and interfacial interactions. Surface roughness can be classified as anisotropic or isotropic, which affects flow directionality and shear stress distribution. Anisotropic textures tend to induce directional fluid transport, whereas isotropic surfaces lead to more uniform but less directed flow behavior. Such effects have been reported to alter lubrication and interfacial flow characteristics [29]. Therefore, surface texture should be considered in analyzing fluid–solid interactions in CMP processes.

Based on the above, combined with the structural characteristics of 40Cr gears and the core principle of traditional CMP technology, this paper proposes a new immersed chemical-mechanical polishing process for gears, aiming to replace traditional mechanical polishing and solve the problems of poor versatility and machining scratches in traditional polishing processes. Three CMP process schemes with different fluid motion forms are designed, including immersed rotation, axial movement, and spiral motion. FLUENT software ANSYS Workbench 2021 (R21) is used to carry out liquid–solid two-phase flow simulation analysis to explore the influence of key parameters such as rotation speed, abrasive particle size, and abrasive concentration on the pressure distribution on the gear surface, and determine the optimal polishing process parameters. The feasibility of the new immersed CMP process is verified through process experiments, providing a new idea for the ultra-precision machining of complex structural parts.

## 2. Principle of Immersed CMP Process

In this study, a certain type of spiral bevel gear is taken as the polishing object. Its key structural parameters are as follows: reference circle diameter 60 mm, module 2, number of teeth 30, face width 15 mm, helix angle 35°, and workpiece material 40Cr high-quality carbon structural steel. The polishing target covers the external surfaces of the gear including the tooth surface, addendum circle, and dedendum circle, aiming to significantly reduce machining traces, obtain a bright and smooth surface, and greatly lower surface roughness.

**Figure 1:**
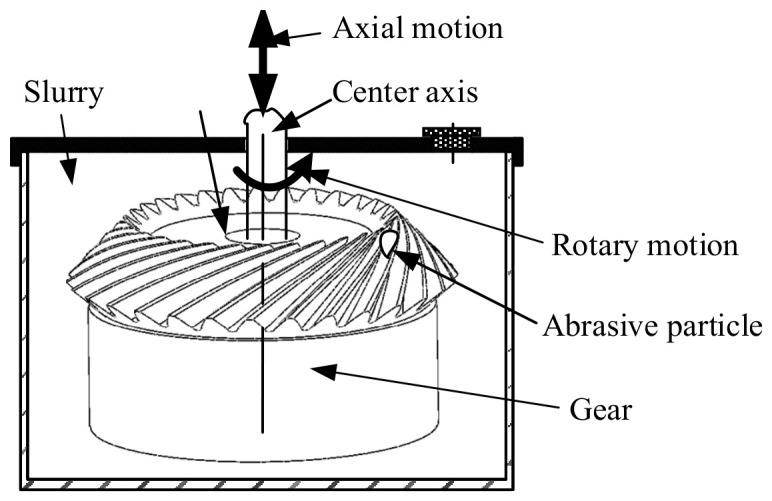
Schematic diagram of the immersion CMP process for gear.

As shown in Figure 1, it is the schematic diagram of the immersed CMP principle for gear. The gear is fully immersed in the prepared polishing slurry to achieve full-surface polishing of the gear tooth profile. The core principle is as follows: the chemical liquid in the polishing slurry undergoes an oxidation and corrosion reaction with the 40Cr gear surface, forming a soft and easily removable chemical reaction softening layer on the tooth surface. By driving the gear to move in a specific pattern in the polishing slurry, turbulent flow of the slurry is induced. The fluid shear force drives solid abrasive particles to perform gentle mechanical micro-cutting on the softening layer of the tooth surface, realizing uniform removal of the softening layer. Meanwhile, the turbulently flowing polishing slurry can promptly carry away polishing debris and continuously renew the contact between the tooth surface and fresh chemical liquid and abrasives, forming a dynamic cycle of “chemical corrosion-mechanical removal-debris discharge”. Ultimately, ultra-smooth and low-damage polishing of the gear surface is accomplished.

According to the rotary structural characteristics of gears and the principle of immersed CMP, three polishing process schemes with different gear motion forms are designed. The motion features of each scheme are listed below:

(a) Immersed rotating immersed CMP: The gear only rotates around its central axis without axial displacement, which is the most basic immersed CMP process with low process control difficulty.
(b) Immersed axial moving immersed CMP: The gear only performs reciprocating axial movement along its central axis without rotation.
(c) Immersed spiral motion immersed CMP: The gear performs both rotation around the central axis and axial movement along the central axis, forming a spiral compound motion.

The motion pattern of the gear directly affects the trajectory of the fluid flow of the polishing slurry and the distribution of shear stress on the gear tooth surface. The fluid flow trajectory envelope corresponds to the polished gear surface, and its coverage completeness determines the tooth surface forming accuracy. Wall shear forces are the main driving force for material removal from the gear tooth surface, and their magnitude and uniform distribution directly affect material removal efficiency and consistency. Therefore, simulation analysis is required to determine the optimal gear motion pattern and process parameters.

**Figure 2:**
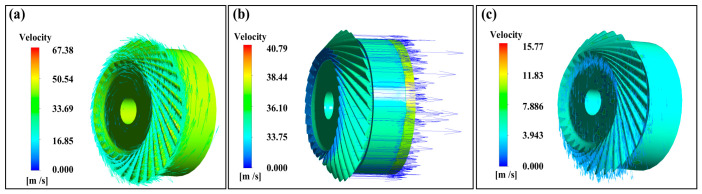
Velocity and trajectory distributions of abrasive particles under different polishing motion conditions: (**a**) immersed rotation, (**b**) axial movement, and (**c**) spiral motion.

The simulation for Figure 2 was conducted using the Euler–Euler two-phase flow model combined with the RNG k–ε turbulence model. The polishing slurry was treated as a liquid–solid multiphase flow system, where abrasive particles were modeled as a dispersed solid phase.

The inlet boundary condition was set as a velocity inlet with an inlet velocity of 0.5 m/s and a turbulence intensity of 5%. The outlet was defined as a pressure-free outflow boundary. The gear surface was modeled as a moving wall with prescribed motion, corresponding to rotational, axial, and spiral motion modes. No-slip boundary conditions were applied to all solid walls.

**Figure 3:**
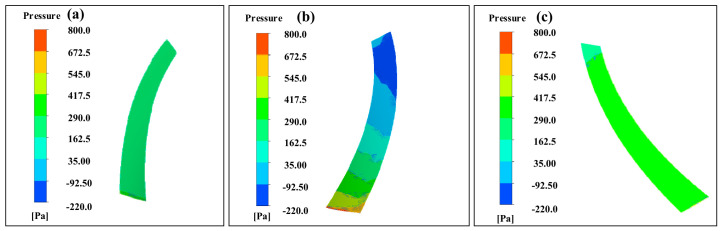
Stress distribution of a single tooth profile under different polishing motion conditions: (**a**) immersed rotation, (**b**) axial movement, and (**c**) spiral motion.

Figure 2 shows the velocity trajectory distribution of abrasive particles under different polishing motion conditions: (a) rotational motion, (b) axial movement, (c) spiral motion. Figure 3 illustrates the contour of stress distribution on a single tooth profile surface. The purpose of this simulation was to compare the particle trajectory distributions under different gear motion modes while keeping all other parameters constant, in order to analyze the influence of motion patterns on slurry flow behavior and surface interaction characteristics.

It can be seen from Figure 2a that when the gear performs rotational motion, abrasive particles move closely along the tooth profile surface, and the stress on each part of the tooth surface is uniform (Figure 3a). From Figure 2b, when the gear performs axial movement, abrasive particles concentrate on the addendum, leading to uneven stress on the tooth profile surface (Figure 3b). From Figure 2c, when the gear performs spiral motion, the trajectory of abrasive particles does not fit closely with the tooth profile surface, resulting in poor uniformity of abrasive particle movement. Moreover, the stress on the small end face is markedly lower than that on the large end face (Figure 3c). In addition, interference occurs in both axial movement and spiral motion in actual polishing due to high abrasive concentration. Therefore, rotational motion is the most suitable.

## 3. Process Simulation and Optimization

### 3.1. Establishment of Simulation Calculation Model

The polishing process of gear immersed CMP is a typical liquid–solid two-phase flow, in which the liquid phase is the chemical polishing fluid and the solid phase is abrasive particles. Since the solid–liquid volume ratio exceeds 10% and the solid phase fraction is significant and cannot be neglected, the solid abrasive particles are treated as a pseudo-fluid in the simulation. Considering both simulation accuracy and computational efficiency, the Euler–Euler method is adopted as the numerical solution for liquid–solid two-phase flow, which can simultaneously describe the spatial distribution and motion of both liquid and solid phases and is suitable for two-phase flow with high solid volume fraction. The RNG k-ε turbulence model is used for turbulent flow calculation, which has strong adaptability to turbulent flows and can accurately simulate the turbulent characteristics of polishing slurry and the wall shear stress distribution on the tooth surface.

Based on the Eulerian model for liquid–solid two-phase flow, the core governing equations of mass conservation and momentum conservation during polishing are established as follows:

**(1)**  **Governing Equations for Liquid Phase**

Continuity equation:

(1) $$\frac{\partial }{\partial t}(\alpha_{l}\rho_{l})+\nabla \cdot (\alpha_{l}\rho_{l}{\vec{u}}_{l})=0$$

Momentum equation:

(2) $$\frac{\partial }{\partial t}(\alpha_{l}\rho_{l}{\vec{u}}_{l})+\nabla \cdot (\alpha_{l}\rho_{l}{\vec{u}}_{l}{\vec{u}}_{l})=-\alpha_{l}\nabla p+\nabla \cdot (\alpha_{l}\mu_{l}\nabla {\vec{u}}_{l})+\alpha_{l}\rho_{l}\vec{g}+M_{ls}$$

Turbulent kinetic energy k equation for RNG k-ε model:

(3) $$\frac{\partial }{\partial t}(\rho_{l}k)+\nabla \cdot (\rho_{l}{\vec{u}}_{l}k)=\nabla \cdot (\alpha_{l}\mu_{eff}\nabla k)+G_{k}-\rho_{l}\varepsilon$$

Turbulent dissipation rate ε equation for RNG k-ε model:

(4) $$\frac{\partial }{\partial t}(\rho_{l}\varepsilon )+\nabla \cdot (\rho_{l}{\vec{u}}_{l}\varepsilon )=\nabla \cdot (\alpha_{l}\mu_{eff}\nabla \varepsilon )+\frac{C_{1\varepsilon }\varepsilon }{k}G_{k}-C_{2\varepsilon }\rho_{l}\frac{\varepsilon^{2}}{k}$$

where *α_l_* is the liquid volume fraction; *ρ_l_* is the liquid density; *u*_l_ is the liquid velocity vector; *p* is the pressure; *μ_l_* is the liquid dynamic viscosity; *g* is the gravitational acceleration; *M_ls_* is the interphase force between solid and liquid; *μ_eff_* is the effective viscosity; *G_k_* is the turbulent kinetic energy produced by the gradient of mean velocity; *C*_1*ε*_ = 1.44 and *C*_2*ε*_ = 1.92 are empirical constants.

The mass conservation equation ensures the continuity of each phase in the immersed CMP process and describes the transport of phase volume fraction in the flow field. The momentum conservation equation represents the force balance acting on each phase. The pressure gradient term drives slurry motion, the viscous term describes internal fluid resistance, and the gravity term accounts for density-driven effects. The interphase momentum exchange term reflects the interaction between abrasive particles, polishing fluid, and the gear surface. These governing equations provide a theoretical basis for analyzing slurry flow behavior and material removal mechanisms in immersed CMP.

**(2)**  **Governing Equations for Solid Phase**

Continuity equation:

(5) $$\frac{\partial }{\partial t}(\alpha_{s}\rho_{s})+\nabla \cdot (\alpha_{s}\rho_{s}{\vec{u}}_{s})+\nabla \cdot (\alpha_{s}\rho_{s}{\vec{u}}_{sd})=0$$

Momentum equation:

(6) $$\frac{\partial }{\partial t}(\alpha_{s}\rho_{s}{\vec{u}}_{s})+\nabla \cdot (\alpha_{s}\rho_{s}{\vec{u}}_{s}{\vec{u}}_{s})=-\alpha_{s}\nabla p+\nabla \cdot (\alpha_{s}\mu_{s}\nabla {\vec{u}}_{s})+\alpha_{s}\rho_{s}\vec{g}+M_{sl}$$

where *μ*_s_ is the solid viscosity coefficient; *M_s_*_l_ is the interphase force between liquid and solid, satisfying *M_sl_* = −*M_ls_*; The solid viscosity is closed by the Ap algebraic model: *μ_s_* = *αsρsτt k*/*ε*, where *τ_t_* is the turbulent fluctuation time of the fluid.

### 3.2. Setting of Simulation Parameters and Boundary Conditions

To ensure good comparability of simulation results under different process parameters, the control variable method is used in this study. Only one variable is changed while keeping the boundary conditions and initial parameters constant.

A numerical model is built in FLUENT. The inlet is set as velocity inlet, with initial liquid and solid velocity of 0.5 m/s, turbulence intensity of 5%, and turbulence viscosity of 0.001 kg/(m·s). The outlet is set as free outflow boundary without additional pressure and velocity constraints. The gear wall is set as a rotating wall with adjustable speed, and the fluid domain walls are fixed no-slip walls.

For pressure–velocity coupling, the Phase-Coupled SIMPLE algorithm is adopted. Spatial discretization uses the second-order upwind scheme. The convergence residual is set to 10^−4^ to ensure accuracy and stability. Simulations are carried out with gear rotation speed (40–100 rpm), abrasive particle size (0.4–0.7 mm), and abrasive concentration (40–70%) as variables to investigate their influence on the pressure distribution on the gear surface.

The selection of simulation parameters is based on the actual immersed CMP experimental system, equipment operating limits, and typical parameter ranges reported in previous studies [30,31]. The inlet velocity, turbulence intensity, slurry properties, and gear rotation speed were set to match real polishing conditions. Therefore, the simulation model reflects practical operating conditions and ensures physical relevance of the results.

### 3.3. Simulation Results and Analysis

The uniformity of pressure distribution on the gear surface is taken as the core evaluation index. Non-uniform stress on the tooth surface tends to cause over-polishing or under-polishing, which seriously affects the consistency of polishing quality. Therefore, uneven pressure distribution must be avoided on the premise of ensuring polishing efficiency.

**(1)**  **Influence of Gear Rotation Speed on Surface Pressure Distribution**

**Figure 4:**
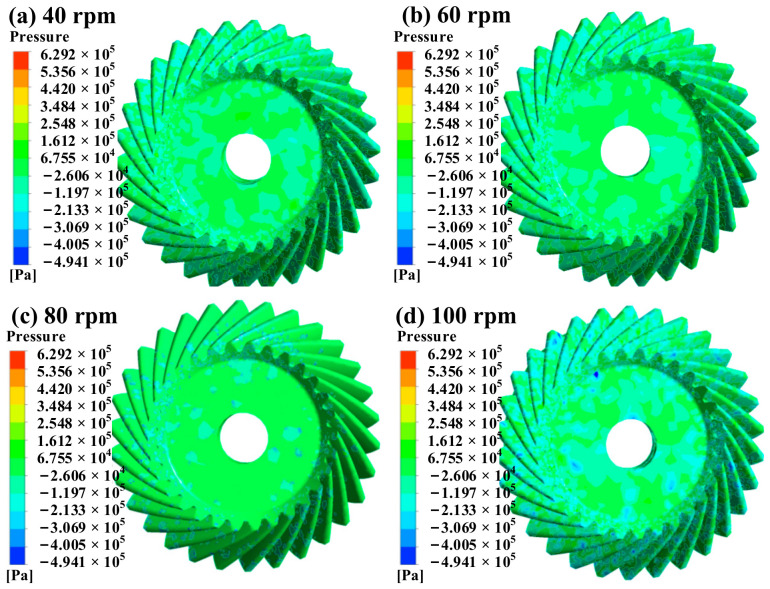
Contour maps of the stress distribution on the gear surface under different rotational speeds.

Figure 4 shows the pressure contour on the gear surface at different speeds, (a) 40 rpm, (b) 60 rpm, (c) 80 rpm, (d) 100 rpm, with abrasive particle size of 0.4 mm and abrasive concentration of 45%.

Simulation results show that at 40 rpm and 60 rpm, the pressure difference between different regions of the tooth surface is large, with small compressive and tensile stresses. At 100 rpm, the pressure distribution becomes more uneven with a larger gradient. At 80 rpm, the overall pressure distribution on the gear surface is relatively uniform with a small pressure difference. The pressure distribution determines material removal during polishing. Large pressure fluctuations lead to inconsistent removal and unwanted waviness. Thus, 80 rpm provides the most uniform material removal and best surface consistency.

**(2)**  **Influence of Abrasive Particle Size on Surface Pressure Distribution**

**Figure 5:**
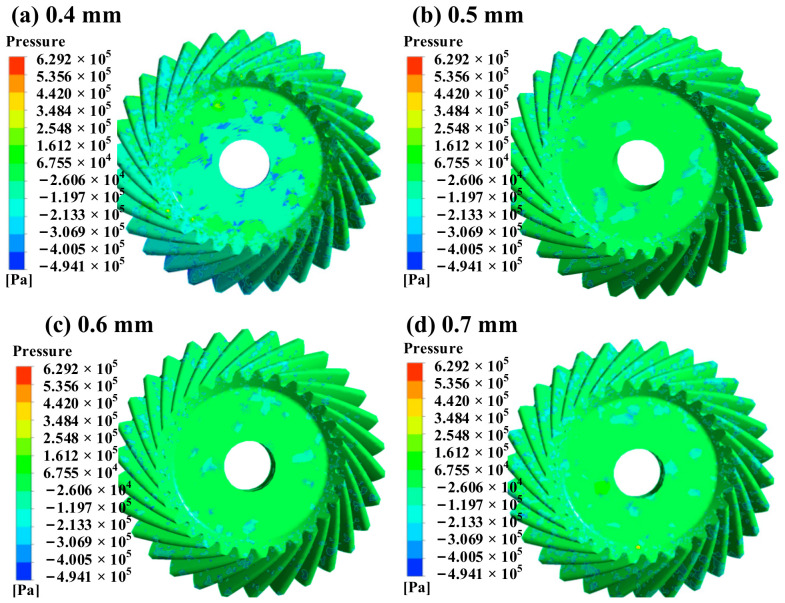
Contour maps of the stress distribution on the gear surface under different abrasive particle sizes.

Figure 5 presents the pressure contours at different abrasive particle sizes, (a) 0.4 mm, (b) 0.5 mm, (c) 0.6 mm, (d) 0.7 mm, with rotation speed of 80 rpm and concentration of 45%.

Results indicate that particle sizes of 0.4 mm and 0.7 mm lead to obvious pressure gradients near the tooth profile edge, which is harmful to uniform material removal. A particle size of 0.5 mm improves pressure uniformity to some extent. A particle size of 0.6 mm results in dominant tensile stress and relatively uniform stress over the whole tooth profile. Therefore, an abrasive particle size of 0.6 mm yields better polishing quality.

**(3)**  **Influence of Abrasive Concentration on Surface Pressure Distribution**

**Figure 6:**
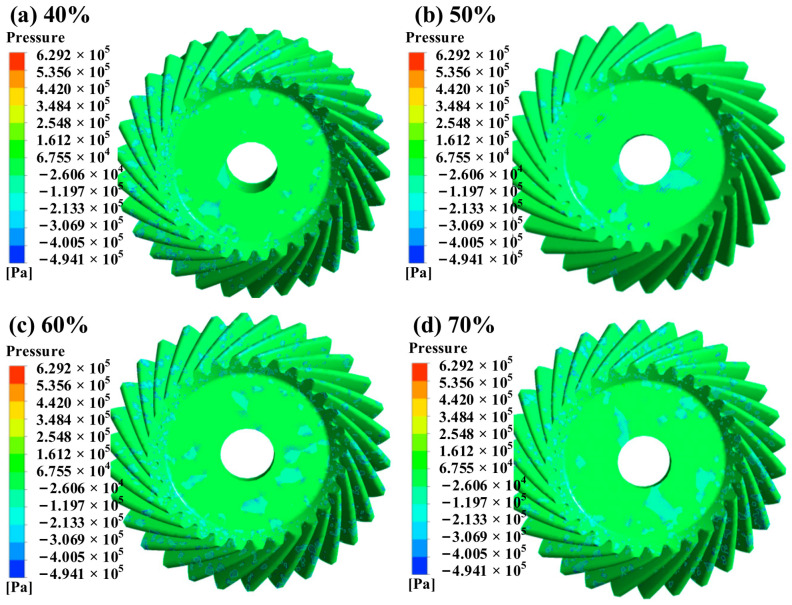
Contour maps of the stress distribution on the gear surface under different abrasive concentrations.

Figure 6 shows the pressure contours at different abrasive concentrations, (a) 40%, (b) 50%, (c) 60%, (d) 70%, with particle size of 0.6 mm and rotation speed of 80 rpm.

It can be concluded that at 50% abrasive concentration, the liquid–solid two-phase flow shows the best fluidity. The turbulent flow of slurry on the tooth surface is uniform, with no obvious pressure gradient and consistent polishing force. When concentration is below or above 50%, the imbalance between solid and liquid phases changes slurry viscosity and fluidity, causing abrasive retention and local pressure concentration. Thus, 50% abrasive concentration ensures uniform stress and optimal surface quality.

Through single-factor simulation analysis of gear rotation speed, abrasive particle size, and abrasive concentration, the optimal parameter combination is determined as: abrasive particle size 0.6 mm, gear rotation speed 80 rpm, abrasive concentration 50%. Under this parameter combination, the turbulent flow characteristics of the polishing slurry and the micro-cutting effect of abrasives achieve the best matching, and the pressure distribution on each region of the gear surface is uniform, which can guarantee the consistency of material removal on the tooth surface and provide reliable parameter support for subsequent process experiments.

## 4. Polishing Experiment

A self-built rotary polishing device was used to conduct immersed CMP on 40Cr spiral bevel gears. The gear parameters are reference circle diameter 60 mm, module 2, number of teeth 30, helix angle 35°, and the gear was prefabricated by CNC milling.

According to the optimal parameters obtained from simulation and relevant references [30,31] on polishing slurry for metal CMP, the processing parameters were set as follows: rotation speed 80 rpm, polishing time 40 min. The polishing slurry mainly consists of 0.5 wt% H_2_O_2_ and 50% concentration silicon carbide abrasives with a particle size of 0.6 mm. Citric acid was used to adjust the pH of the slurry to 4, and the H_2_O_2_ solution was prepared using a 30% hydrogen peroxide stock solution.

**Figure 7:**
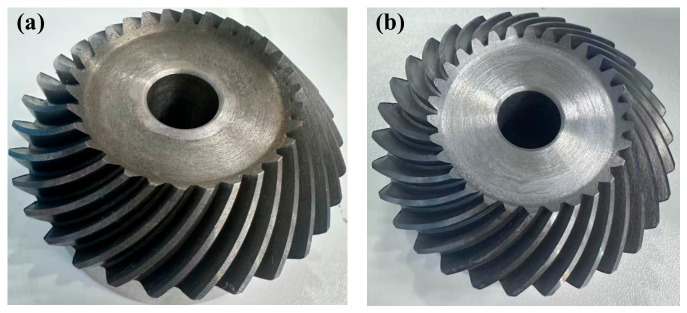
Overall physical photographs of the spiral bevel gear: (**a**) before polishing, and (**b**) after polishing.

**Figure 8:**
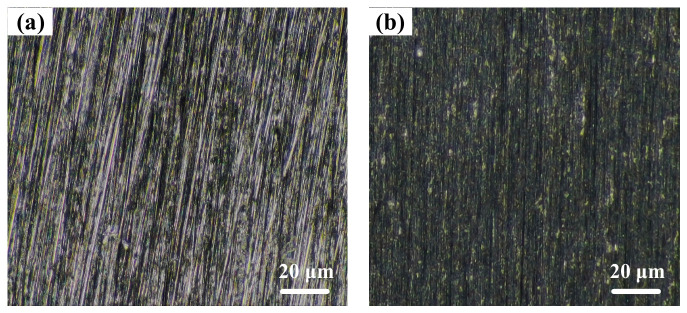
Surface morphology of the spiral bevel gear: (**a**) original surface, and (**b**) polished surface.

The surface micromorphology and roughness of the gear before and after polishing were characterized by a metallographic microscope and TR200 handheld roughness tester. Figure 7 shows the physical comparison of the spiral bevel gear before and after polishing. As shown in Figure 8, obvious machining scratches and surface defects can be observed on the tooth surface before polishing. After immersed CMP, the surface scratches are significantly reduced, and the tooth surface becomes smoother.

**Figure 9:**
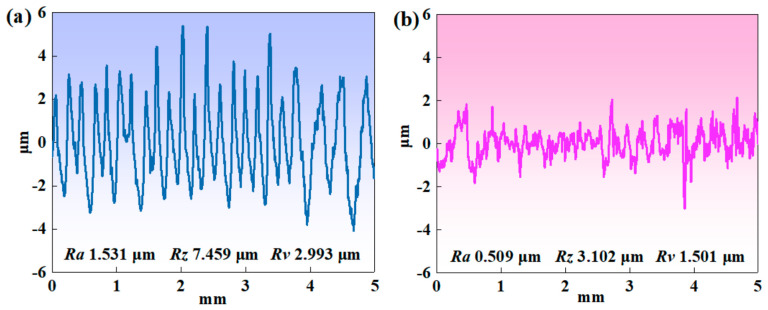
Surface profile curves of the gear tooth surface: (**a**) before polishing, and (**b**) after polishing.

The curves in Figure 9 represent the surface profile variation along the measured tooth profile direction. Before polishing, the curve shows large fluctuations, indicating the presence of machining scratches and surface waviness. After immersed CMP, the fluctuation amplitude is significantly reduced. The surface roughness is Ra 1.531 μm, Rz 7.459 μm, Rv 2.993 μm. After polishing, the workpiece becomes bright and smooth, scratches are greatly reduced, and the surface roughness is decreased to Ra 0.509 μm, Rz 3.102 μm, Rv 1.501 μm.

The “optimal result” obtained in this study represents a local optimum within the investigated parameter range. Other nearby parameter combinations may also yield comparable performance under different evaluation criteria. Future work will focus on multi-factor optimization and experimental validation to further improve and verify the robustness of the optimal parameters.

## 5. Conclusions

Aiming at the technical bottleneck of high-quality surface machining for 40Cr gears, this study proposes a novel immersed chemical-mechanical polishing process. Based on FLUENT simulation optimization and experimental verification, the main conclusions are as follows:

(1) Three CMP process schemes were designed, including immersed rotation, axial movement and spiral motion. The influence of gear motion modes on the flow trajectory of polishing slurry and wall shear stress of tooth surface was clarified, which provides a reference for CMP process design of complex-structured gears.
(2) A liquid–solid two-phase flow simulation model for gear immersed CMP was established using the Euler–Euler method and RNG k-ε model. The effects of rotation speed, abrasive particle size and concentration on tooth surface pressure distribution were studied via the control variable method. The optimal process parameters are determined as abrasive particle size 0.6 mm, gear rotation speed 80 rpm, abrasive concentration 50%, under which the pressure distribution on the tooth surface is uniform.
(3) Experiments were carried out on a self-built polishing device. After 40 min of polishing under optimal parameters, the scratches on 40Cr gear surfaces are significantly eliminated, and the surface roughness Ra is reduced from 1.531 μm to 0.509 μm. The tooth surface quality is effectively improved.

This work provides a feasible and efficient technical solution for precision polishing of complex gear structures and broadens the application of CMP technology to non-planar component machining. However, the present study still has some limitations. The coupling effects among process parameters were not fully considered, and the chemical reaction mechanism was simplified. Future work will focus on multi-factor optimization, improved fluid chemical mechanical coupling models, and validation on different gear geometries.

## Data Availability

Data are contained within the article.
